# Non-invasive treatment for severe complex pressure ulcers complicated by necrotizing fasciitis: a case report

**DOI:** 10.1186/s13256-015-0703-8

**Published:** 2015-09-18

**Authors:** Geng-jia Tian, Ying Guo, Li Zhang

**Affiliations:** Chronic Wound Treatment Center, Hangzhou Geriatric Hospital, Hangzhou, 310012 Zhejiang China

**Keywords:** Necrotizing fasciitis, Negative pressure wound therapy with instillation, Pressure ulcers

## Abstract

**Introduction:**

Pressure ulceration is a common problem for long-term bedridden patients and individuals with traumatic paraplegia. Necrotizing fasciitis can be a life-threatening complication caused by pressure ulcers, especially for debilitated elderly patients. In this report, we describe the successful use of negative pressure wound therapy with instillation to treat severe complex pressure ulcers complicated with peri-anal necrotizing fasciitis.

**Case presentation:**

A 58-year-old Chinese woman was admitted to our hospital with severe complex pressure ulcers on her bilateral ischial tuberosities, left hip, perineum, and left sacrococcygeal region. The wounds had been present for nearly 2 years. Her seventh and eighth thoracic vertebrae had been traumatically injured; she had been bedridden for 5 years. She was also diabetic. Her medical history and laboratory investigations confirmed severe complex pressure ulcers complicated with necrotizing fasciitis. Antibiotic therapy was initiated. Following negative pressure wound treatment with instillation, the topical infection subsided and final closure of the wound occurred after 130 days.

**Conclusions:**

Negative pressure wound treatment with instillation is an effective treatment protocol. It can reduce healing time, and promote long-term functional and cosmetic outcomes in debilitated patients with severe complex pressure ulcers complicated with necrotizing fasciitis.

## Introduction

Necrotizing fasciitis (NF) is caused by serious bacterial infection, which spreads rapidly and destroys the body’s soft tissue and fascia planes. In the elderly, it often occurs because of severe complex sacral pressure ulcers. Peri-anal NF generally involves the crissum and perineum trigonum; this usually results in septicopyemia, shock and even death [1]. The mortality rate can range from 25 to 45% [2]. The problematic wound presents major challenges in terms of reconstructive options and surgical management outcomes.

## Case presentation

Our case was a 58-year-old Chinese woman who had presented with severe complex pressure ulcers 2 years earlier. She was an obese (body mass index, BMI, 30.05kg/m^2^) paraplegic who presented with fever and anemia; she was admitted from home to the Chronic Wound Treatment Center in July 2014. Her past medical history included type 2 diabetes mellitus (managed with insulin), hypertension, and chronic gastrointestinal dysfunction. She had developed paraplegia following a traumatic injury 40 years earlier. It was reported that a wound on her ischial tuberosities had started to produce purulent secretions, together with labia majora redness 12 days earlier. It was observed that she had pyrexia and labia majora ulceration 2 days before hospitalization.

On admission, a physical examination revealed a high fever (39.2°C) and lower limb edema. Her heart rate was 104 beats per minute (bpm) and blood pressure was 110/80mmHg (after medication). Her neurological examination was unremarkable expect for hypermyotonia in her lower limbs. A continuous indwelling urethral catheter was used because of an absence of lower limb sensations.

Multiple ulcers covered with necrotic tissue were located on her bilateral ischial tuberosities, left hip, perineum, and left sacrococcygeal region. Local skin examination of these sites revealed an absence of pain, and no exudates were observed. No regional lymphadenopathy was noted. However, there were clear signs of infection and purulence with redness extending several centimeters from the coxal wound margins to the genital region, which had weeping hemoserous fluids.

A computed tomography scan of her hip at a previous admission to another hospital 22 days later, showed a lesion with rarefaction areas in her sacral area, and an obvious gas shadow on her left buttock, manifesting itself as a soft tissue infection on her left hip and ischium. Laboratory tests revealed a high leucocyte count level (white blood cell, WBC =21×10^9^/L), high C-reactive protein level (CRP=189.6mg/L), slight anemia (hemoglobin, HGB =96g/L), and low serum potassium level (K=3.26mmol/L). Her urine culture confirmed infection with *Citrobacter freundii*. Swabs from the pressure ulcers were cultured; *Escherichia coli* were isolated from the culture and were found to be sensitive to amikacin. The rest of the laboratory tests (other electrolytes, blood culture) were within normal values.

She was initially treated with mezlocillin sodium (6g per day) followed by levofloxacin (0.6g daily) for 2 weeks, both via intravenous administration. She was also treated with oral montmorillonite powder (once every 3 to 5 days) to control her bowel movements.

Without anesthesia, a conservative sharp debridement of the wound bed was carried out. To clean the wound with widely undermined areas, we inserted a sterile silicone tube (an irrigating tube of 6mm diameter) deep into the tunnels and then applied the areas with 3% hydrogen peroxide, iodine, 0.5% metronidazole, 0.9% sodium chloride solution, one after another, using a 50ml syringe.

**Fig. 1:**
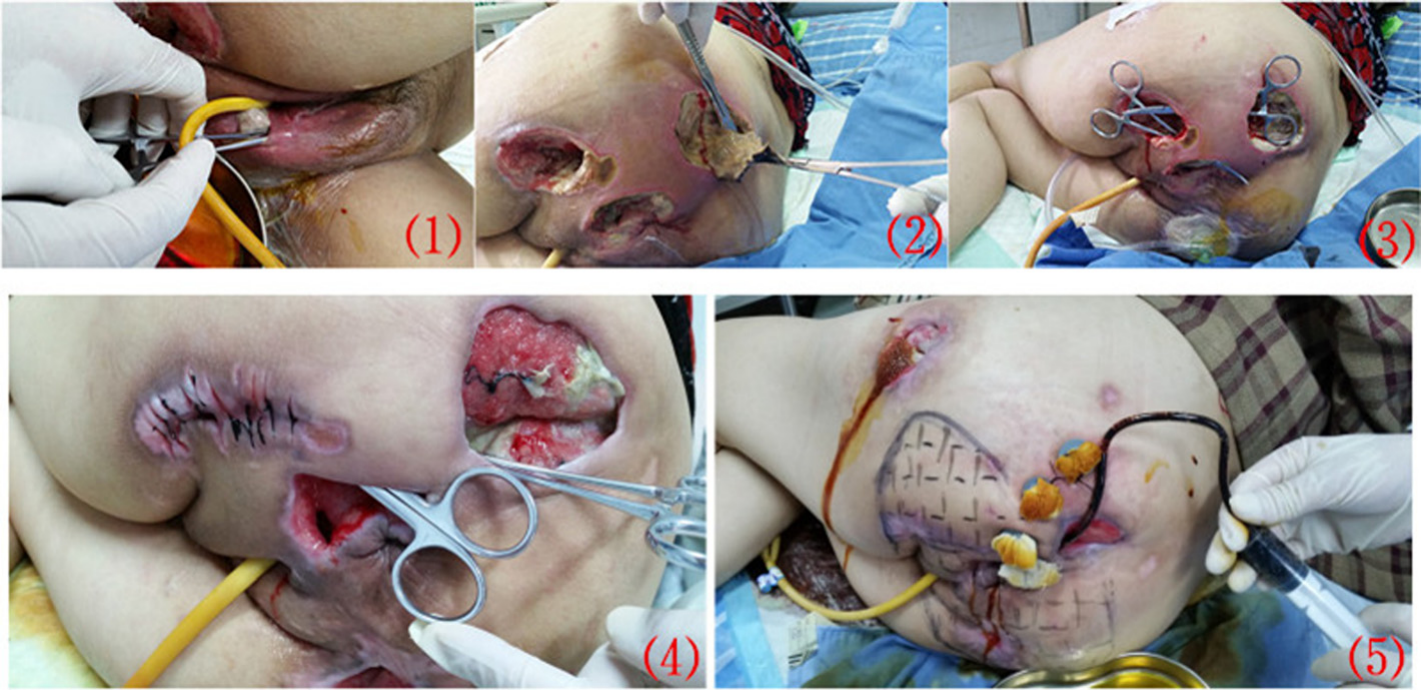
Severe complex pressure ulcers complicated by necrotizing fasciitis. (1)-(3) Initial clinical aspect of the severe complex pressure ulcers; (4) Wound at 8 weeks; (5) Wound at 13 weeks

An accurate assessment of the wound area was made by conducting a thorough evaluation of the size, depth, presence of granulation tissue, wound edges, skin temperature, and exudates and/or necrosis. Large ulcers were present in the ischial tuberosities (size: left, 6.0×7.0cm; right, 4.0×5.0cm, both with undermining), left hip (size: 5.0×6.0cm with undermining), left sacrococcygeal region (size: 10.0×9.0cm with undermining) and genital region (with undermining). The tunnels ranged from 8.0 to 20cm in length. All of the open wounds communicated with each other within the deep soft tissues and were close to the anus (see Fig. 1). All pressure ulcers were at stage IV with eschar or slough.

**Fig. 2:**
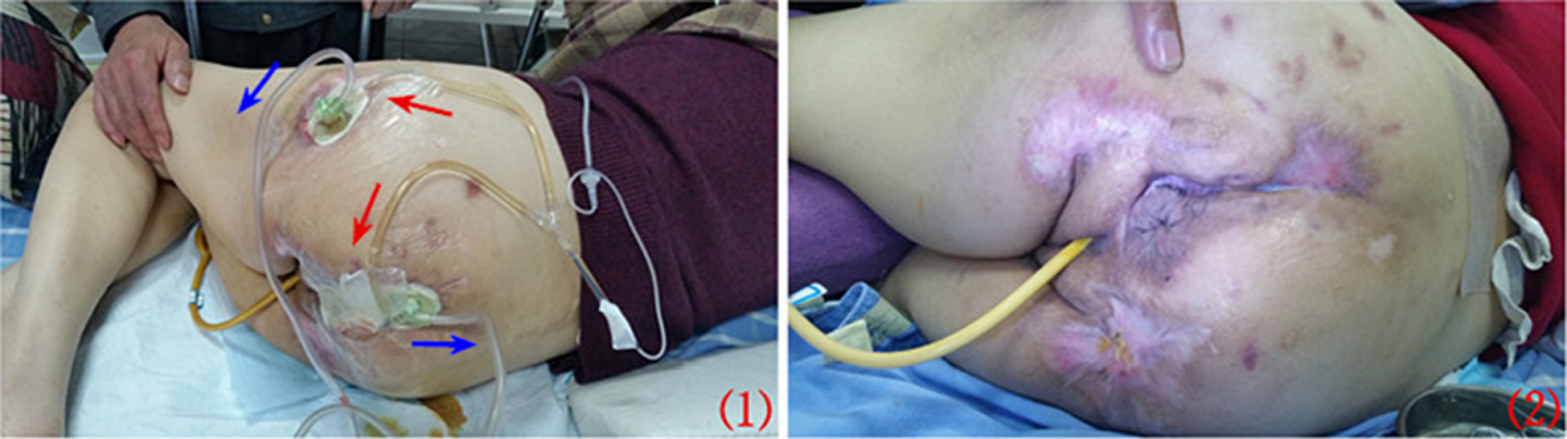
(1) Negative pressure wound therapy with instillation (the red arrows indicate irrigation tubes and the blue arrows indicate the drainage tubes); (2) Wound healing

After cleansing the wounds and preparing the peri-wound areas, the wound drain tubes were laid on top of foam; the irrigating tube (with lateral perforations) was then inserted inside and deep into the tunnel. The negative pressure therapy used polyurethane foam and wall suction. Negative pressure wound therapy with instillation (NPWTi; see Fig. 2-1) was initiated immediately: 5% povidone iodine (10ml) and normal saline (500ml) was instilled twice a day. Instillation was repeated every 24 hours by continuous negative pressure at −80mmHg.

Proliferation of healthy granulation tissue was observed with a decrease in scores of pressure ulcer severity, which was measured every 5 to 7 days; complete re-epithelialization was eventually achieved (see Fig. 2-2). The wounds healed within 130 days of admission without any severe complications. No additional treatment for this patient is anticipated, except for ​petroleum jelly, which will be applied to protect the restoring wound area.

Three months after discharge, the patient was reviewed using a one-to-one interview. She did not have any new occurrences or recurrence of pressure ulcers.

## Discussion

In this case report, we have described our experience and long-term follow-up results of applying NPWTi to pressure ulcers that were not suitable for conventional and/or surgical treatments. It should be emphasized that negative pressure wound therapy (NPWT) was effective and instillation achieved pleasing results. Our findings show the granulation can be harvested from wound areas without any major complications or the need for aggressive procedures. In addition, ideal cosmetic and functional results can also be achieved.

The etiology of pressure ulcers includes pressure, friction, shearing forces, nutritional status, social status, psychological status, and underlying disease. A classification system of pressure ulcers has been developed previously, and the surgical management of these lesions has also been reviewed. Infections secondary to pressure ulcers are usually limited to patients who are domiciled in their homes. In our study, the patient developed ulceration in her genital region, which is not commonly affected by pressure ulcers. We concluded that these infections resulted from loose peri-anal connective tissue and fasciae, which were worsened by the patient living at home.

The current treatment options for pressure ulcers mainly include dressing, negative pressure therapy, and surgical operations. Standard NF treatments involve surgical debridement, vascularized tissue, intravenous antibiotic therapy, hyperbaric oxygen therapy, and wound care. However, dressing often results in poor tolerance and prolonged length of stay. Operations are often not suitable for debilitated patients, such as the aged. Soft tissue reconstruction on the sacral, coccyx and crissum is a challenging problem for reconstructive surgeons because of poor tolerance, challenging surgical debridement, and limited flap alternatives. When accompanied with complex tissue damage following major injuries, local flap alternatives are not available and reconstruction can be difficult.

Wounds should always be approached as complex injuries that are contaminated with a large number of microorganisms, which may delay wound healing and proliferation. Death from overwhelming systemic infection can occur.

NPWT systems work by inducing positive pressure at the wound bed. This creates a zone of hypoxic tissue local to the wound bed, which is surrounded by a zone of hyperemia in the peri-wound tissue. Neumaier J [3] reported NPWT to be the recommended approach for treating heavily exuding wounds because negative pressure systems facilitate the removal of interstitial fluids. QU YS* et al.*[4]noted NPWT prevents inflammation. Patmo AS *et al.* [5] reported vacuum-assisted closure usage can decrease bacterial load in wounds. In addition, following NPWT, granulation tissue is promoted and an improved blood supply can be detected at the site. According to Gabriel *et al*. [6], NPWT accelerates debridement in a similar fashion to autolyzed debridement and physical debridement. It can also stimulate biofactors that can further promote wound healing. Gabriel *et al*. [6] suggested (p. 405) that NPWT combined with topical irrigation after debridement is superior to standard NPWT treatment for infection control; this may decrease would healing times, duration of hospital stay, and length of therapy.

Irrigation solutions have several alternatives. Matiasek *et al*. [7] reported a case study that combined NPWT with instillation using an octenidine-based wound rinsing solution. In this study, NPWT was combined with a povidone iodine-based wound irrigating solution. Moore and Cowman [8] concluded that there was insufficient trial evidence to support the use of any particular wound cleansing solution or technique for managing pressure ulcers. Normal saline is favored as it is an isotonic solution and does not interfere with the normal healing process.

New occurrences and the recurrence of pressure ulcers within 2 years of treatment can be as high as 42.4% in our hospital. We have observed that healed wounds result in positive cosmetic and functional outcomes. We believe that NPWTi can accelerate early closure of wounds and it can also play a role in reducing recurrence rates post-discharge for up to several years.

## Conclusions

When not contraindicated, NPWTi is a safe and effective modality for treating pressure ulcers with tunnels and undermines. It is also a promising therapeutic alternative for debilitated patients with severe complex pressure ulcers complicated with NF.

## Consent

Written informed consent was obtained from the patient for publication of this case report and accompanying images. A copy of the written consent is available for review by the Editor-in-Chief of this journal.

## Abbreviations

NF: Necrotizing fasciitis
NPWT: Negative pressure wound therapy
NPWTi: Negative pressure wound therapy with instillation

## References

[1] Chen WP, Feng HL, Shen YT, Mao KR. Diagnosis and treatment of perianal necrotizing fasciitis: 5 cases report. J Colorectal Anal Surg. 2011;17(6):392–3.

[2] Li S, Sun ZF. Acute necrotizing fasciitis caused by perianal abscess: 1 case report. Chin Health Care Nutr. 2014;24(5):2538–9.

[3] Neumaier J. Innovative therapeutic measures in problem wounds: autologous skin and vacuum sealing. MMW Fortsehr Med. 2004;146(17):14. 15224896

[4] Qu YS, Cui ZM, Qu Y. Evaluation of the quality of life of patients with necrotizing fasciitis on treatment of negative pressure wound therapy. Chin J Health Stat. 2013;30(6):936–7.

[5] Patmo AS, Krijnen P, Tuinebreijer W, Breederveld RS. The effect of vacuum-assisted closure on the bacterial load and type of bacteria: a systematic review. Adv Wound Care. 2014;3(5):383–9. 10.1089/wound.2013.0510 PMC4005496 24804158

[6] Gabriel A, Shores J, Heinrich C, Baqai W, Kalina S, Sogioka N, et al. Negative pressure wound therapy with instillation: a pilot study describing a new method for treating infected wounds. Int Wound J. 2008;5(3):399–413. 10.1111/j.1742-481X.2007.00423.x PMC7951189 18593390

[7] Matiasek J, Djedovic G, Mattesich M, Morandi E, Pauzenberger R, Pikula R, et al. The combined use of NPWT and instillation using an octenidine based wound rinsing solution: a case study. J Wound Care. 2014;23(11):590. 592-6. 10.12968/jowc.2014.23.11.590 25375407

[8] Moore ZE, Cowman S. Wound cleansing for pressure ulcers. Cochrane Database Syst Rev. 2013, Issue 3. Art. No. CD004983. doi:10.1002/14651858.CD004983.pub3. PMC7389880 23543538

